# Changes in Eating Habits and Physical Activity after COVID-19 Pandemic Lockdowns in Italy

**DOI:** 10.3390/nu13124522

**Published:** 2021-12-17

**Authors:** Mauro Lombardo, Elena Guseva, Marco Alfonso Perrone, Alexander Müller, Gianluca Rizzo, Maximilian Andreas Storz

**Affiliations:** 1 Department of Human Sciences and Promotion of the Quality of Life, San Raffaele Open University, 00166 Rome, Italy; elena.guseva@uniroma5.it; 2 Division of Cardiology, University of Rome Tor Vergata, 00133 Rome, Italy; marco.perrone@uniroma2.it; 3 Department of Internal Medicine II, Center for Complementary Medicine, Freiburg University Hospital, Faculty of Medicine, University of Freiburg, 79106 Freiburg, Germany; alexander.mueller@uniklinik-freiburg.de (A.M.); maximilian.storz@uniklinik-freiburg.de (M.A.S.); 4 Independent Researcher, Via Venezuela 66, 98121 Messina, Italy; gianlucarizzo@email.it

**Keywords:** food, eating, habits, lifestyle, physical activity, COVID-19

## Abstract

The introduction of lockdowns and other containment measures during the COVID-19 pandemic substantially altered people’s lifestyle and dietary behavior. Several studies evaluated the short-term effects of these measures; yet reports on long-term consequences are scarce. We sought to address this gap in the literature by analyzing dietary and lifestyle data collected at an obesity center in Rome, Italy. The Italian region of Lazio was hit hard by the pandemic. To evaluate the potential health impacts, we compared the pre- and post-lockdown data of 118 individuals. Contrary to the common belief that lockdown had adverse effects solely on people’s dietary habits, we observed a significantly increased consumption of raw vegetables, whole grains, and water in our study sample. Favorable effects, however, were also accompanied by adverse trends, such as a higher prevalence of sleeping difficulties. Our data emphasize that the lockdowns associated with the pandemic also influenced participants’ social behavior, with less individuals reporting eating out or in company. Our study highlights the substantial impact of lockdowns on many dimensions of life. As such, it is of utmost importance in the critical evaluation of such stringent containment measures.

## 1. Introduction

The advent of the COVID-19 pandemic has caused substantial changes in people’s eating and lifestyle behaviors. Due to several consecutive months of physical and social isolation, many individuals modified their dietary habits. Several studies evaluated the short-term effects of lockdown on nutritional habits, emphasizing both positive and negative changes [1,2].

The Italian region of Lazio experienced a tight lockdown phase (between 9 March and 3 May 2020), with a subsequent tightening of containment measures (between 4 May and 14 June 2020). Thereafter, from 15 March 2021 until June 2021, the Italian government again imposed several other strict lockdown periods. The most severe lockdown period was characterized by the suspension of common commercial activities, catering services, religious celebrations, and the banning of people gathering in public places. Regular office work was discontinued for most professions and was replaced by working from home. For students, in-person teaching was suspended [3]. From 6 August 2021, normal activities gradually returned, especially for vaccinated persons with a green pass [4]. The reactivation of activities showed the necessity to deal with new routines and habits [5]. Some studies have shown favorable changes in individuals’ eating habits, with an increase in the number of meals prepared and consumed at home [3,4,5]. Favorable effects were accompanied by other negative ones, such as an increase in alcohol consumption [4], increased “comfort food” consumption due to emotional distress [5], and reduced sleep quality [6]. These changes were accompanied by a more sedentary lifestyle due to increased screen time [7], smart working and the resultant abolition of walking or public transport to work [8], and fewer opportunities for practicing physical activity [9]. Most studies have evaluated the short-term effects of social distancing and home isolation [5,6,7,8,9]. However, there is a lack of studies assessing the long-term effects of quarantine periods. The aim of this study was to evaluate the potential impact of lockdown periods on eating habits and health risk factors through the use of a questionnaire that was administered before and after the lockdown periods.

## 2. Materials and Methods

We collected data related to eating behaviors, food habits, and food taste before and after the aforementioned lockdown periods at an obesity center in Rome, Lazio, Italy. For this study, we defined the pre-lockdown period (“before”) as the time before March 2020. All procedures performed were in accordance with the ethical standards of the 1964 Helsinki Declaration and its later amendments. The study was approved by the Ethics Committee of the University Hospital “Tor Vergata” in Rome (ID number 44.22) and date of approval is 06 April 2020.

The survey was conducted among patients in a nutrition practice who were asked to complete an online questionnaire in the Italian language prior to their first visit. The survey was disseminated through an online form created specifically for this purpose. Altogether, 1256 individuals answered the survey between June 2018 and January 2020. In this sample of subjects, we assessed gender differences in taste and lifestyle habits [10]. The inclusion and exclusion criteria were established before the first survey to reduce the number of external confounders and to ensure a homogenous sample. In summary, patients with age < 12 years or >75 years, pregnant or breastfeeding, or a BMI < 18.5 kg/m^2^ were excluded.

All 1256 participants were contacted via a dedicated newsletter between May and July 2021, with the request to repeat the same online questionnaire. The online survey, which required approximately 30 min to complete, allowed participants to answer the test via any electronic device with internet access. When accessing the link, participants expressed their consent to the use of the data and had the option to consent or not to participate. We recorded all responses anonymously. The online questionnaire consisted of four parts, as described elsewhere in detail [10]. The first part of the survey investigated daily meals, hunger during the day, eating disorders, and sleep quality. The second part of the test asked subjects about the consumption frequency of several food groups individually, with the option to respond (“yes”, “no”, and “rarely”). The third part of the survey inquired about favorite dishes, a typical day’s eating, and the daily consumption of water as well as alcoholic and sugary drinks. The last part was dedicated to physical activity and included number of hours per week devoted to sport, time of day when sport was practiced, and type of sport. Average weekly METs (the ratio of the work metabolic rate to the resting metabolic rate) were calculated using the time spent per week on each sport activity.

An expert registered dietician performed the dietary history, and body composition (BC) assessments were evaluated in the days following the first meeting. Weight and height were determined with the subjects fasting overnight and wearing only underwear. Weight and body composition were assessed from a standing position after overnight fasting using a Tanita BC-420 MA (TANITA Corporation, Sportlife Tokyo, Japan, range of 0–200 kg, accuracy: 100 g), an instrument validated using dual-X-ray absorptiometry (DXA) [11]. Patients were required to observe the following guidelines before BC evaluation: 3 h or more after meals and not eating or drinking too much the day before the measurement; 3 h after awakening and beginning normal daily activities; 12 h or more after a hard workout; urination before the measurement; avoiding alcohol 12 h before the visit. Women were asked to avoid the visit in the days before and during the menstrual cycle because this could affect the accuracy of the measurement. The measurement of body composition in female subjects was performed only during the period from day 6 to day 24 of the menstrual cycle. We considered for this study only two parameters that were measured during the visit: fat mass (FM) and fat-free mass (FFM).

### Statistical Analysis

Statistical analysis was performed using StatTech v. 2.1.0 (Developer: StatTech LLC, Kazan, Russia). Quantitative variables were assessed for normality using the Shapiro-Wilk test (when the number of subjects was less than 50) or the Kolmogorov-Smirnov test (when the number of subjects was more than 50). Quantitative variables following non-normal distribution were described using median (Me) and lower and upper quartiles (Q1–Q3). The Wilcoxon test was used for comparison of quantitative variables following non-normal distribution between two matched samples. In light of the pre-planned study hypothesis, we did not make use of the Bonferroni correction [12].

## 3. Results

A total of 118 participants completed both questionnaires and were included in the final analysis. Table 1 shows the baseline characteristics of all participants.

Our study included 85 females (72%), with a mean age of 44.2 ± 11.8 years. The numbers for the different age groups are 20–30 years old, 15 (12.7%); 31–40 years old, 34 (28.8%); 41–50 years old, 37 (31.4%); 51–60 years old, 19 (16.1%); 61–70 years old, 11 (9.3%), and 71–75 years old, 2 (1.7%). Mean BMI (Body Mass Index) was 26.2 ± 4.8 kg/m^2^. Fifty-seven subjects (48.3%) had a normal weight (BMI = 18.5–25), 41 (34.7%) were overweight (BMI = 25.1–30), 12 (10.2%) had first degree obesity (BMI = 30.1–35), 6 (5.1%) had second degree obesity (BMI = 35.1–40), and 2 (1.7%) had third degree obesity (BMI = 40.1–45). Mean at mass was 22.2 ± 9.6 kg, and mean fat free mass was 48.2 ± 9.7 kg. The different work activities of the subjects are shown in Table S1 included in the supplementary material. The mean physical activity was 7.8 ± 8.4 METs/week. Seven patients suffered from type 2 diabetes mellitus (5.4%), 23 had hypertension (17.8%), and 38 had dyslipidemia (29.5%).

Regarding food group patterns, we observed some significant differences before and after the lockdown periods (Table 2) for the food consumption frequency for cereals (e.g., spelt and barley) (66.9% “before” vs. 76.3% “after”; *p* = 0.008), raw vegetables (78.8% “before” vs. 92.4% “after”; *p* < 0.001) and legumes (94.1% “before” vs. 87.3% “after”; *p* = 0.034). The most pronounced effect was found in raw vegetable consumption (Table 2).

In addition to the frequency of food types, we observed some significant changes in drinking habits among participants before and after the lockdowns (Table 3). We observed no differences with regard to alcohol intake.

Table 4 shows other relevant changes in dietary and sleeping habits and sports frequency before and after lockdowns.

The context in which meals were eaten changed as well. Participants ate out of home less often (24.6% “before” vs. 48.3% “after”; *p* <0.001) and ate lunch less frequently at a company canteen (16% “before” vs. 6.8% “after”; *p* = 0.012). Patients reported an increased frequency of sleeping disorders. We found a 6.7% increase in the number of people who said they had difficulties falling asleep at night (*p* = 0.33). Furthermore, we observed no reduction in time spent for physical activities, even though 22% of the subjects stated that they practiced more sport in the morning, as compared to only 12.7% prior to lockdowns. (*p* = 0.016) Table 2, Table 3 and Table 4 highlight other, non-significant trends prior to and after the lockdown periods.

## 4. Discussion

The long periods of isolation had an impact on patients’ preferences and eating habits. It is commonly believed that lockdown is a period characterized by home-confinement and consumption of unhealthy foods, creating an obesogenic environment [13,14]. On the contrary, for many people, the increased availability of time was an opportunity to improve their eating habits, partly due to greater involvement in the kitchen [15]. Previous studies demonstrated that a lack of willpower, time constraints, and taste preferences are the main difficulties in people eating healthy [16] It is likely that the lockdowns’ substantial impact on people’s life induced significant improvements in several nutritional behaviors. For example, a French study [17] revealed that 83% of patients reported increased time spent cooking during self-quarantine.

In our study, we sought to assess the possible changes in lifestyle habits subsequent to periods of isolation and the particular general situation induced by the pandemic. Confirming the aforementioned studies, our data also show lockdowns were associated with some good eating habits, possibly due to the increased time available for meal preparation.

One example is the increased preference for raw vegetables and whole grains in our sample. Other studies [18,19] have shown that the quality of diets slightly improved and the prevalence of food insecurity was reduced in healthy populations. One potential explanation is that governmental guidance for a healthy diet to prevent COVID-19 was widely followed and implemented by the Italian population. Undoubtedly, the increased time spent at home in our sample of smart workers also contributed to the preference for fresh foods, which often require extensive preparation. It is conceivable that regular office workers would otherwise not have had the time to cook such meals.

Several changes may have been the effect of governmental campaigns to prevent COVID. The World Health Organization recommended legumes, fruits, and vegetables as the best food items during self-quarantine or longer home stays [20]. Our data showed that participants also reported drinking more water as compared to the pre-lockdown period. This could be explained by the increased time spent at home and water consumption being a more health-conscious behavior. Many employees, especially those working in contact with the public, are forced to limit their daily water consumption to avoid having to leave the workplace to use toilets [21]. Smart working probably improved this, as it provides more flexibility in work schedules and better opportunities to take breaks.

The consumption of sugary drinks was reduced as well. At workplaces, snack bars or vending machines are frequently used to sell snacks, fruit juices, and soft drinks. These places also serve as important gathering points for breaks and for interpersonal communication. Working from home may have reduced these habits [22]. As expected, our patients reduced the number of meals outside the home, particularly at lunchtime. At the time the second survey was administered, a large number of employees still worked from home, and access to places to eat outside the home was limited, particularly at lunch time.

It has been previously demonstrated that eating out of the home may be an important risk factor for lower micronutrient and higher energy intake (mainly from fat) [23]. In contrast, a study of adults from 11 European countries showed that eating at work is similar to eating at home. Only the higher consumption of alcoholic beverages was responsible for the higher caloric intake of those who ate mainly in restaurants [24]. However, our data did not detect any substantial increase in alcohol consumption, supporting the first of both aforementioned studies [23].

We observed some changes in the physical activity habits of our participants. Although the type of sport and the amount of time spent on physical activity remained the same, the time of day that sports were practiced changed, with an increase in morning hours. While previous studies suggested a reduced physical activity during lockdowns [25,26], we observed a “return to normal” with regard to sports practice. One explanation is that the more liberal free-time management involved in smart working allowed people to exercise more often in the morning.

Our study sample shows an increased frequency of sleep disturbances, especially in the first phase of sleep (falling asleep). This suggests that the pandemic has had long-lasting and substantial effects, since the last lockdown ended months ago. These data confirm other studies conducted during the COVID-19 pandemic, highlighting increased psychological distress and significant sleep disturbances in many individuals [27,28]. It is likely that the proximity of the most severe period of the pandemic left a legacy of uncertainty and psychological distress in our study sample. It will be interesting for future studies to investigate whether these alterations in sleep quality, which in turn have a great influence on dietary quality [29], remain when the pandemic is completely over.

Our study suffers from several limitations that warrant discussion. Many participants did not agree to repeat the survey a second time; as such, the number of participants is limited to *n* = 118. The questionnaire we used did not consider energy intake, and we did not stratify the sample by BMI. Undoubtedly, the physical state of our participants could have influenced the relationship with food, taste, and eating habits. We also have to consider that some patients had their last dietary examination months before the second survey, which could introduce a certain bias. Finally, all items were self-reported, potentially introducing reporting bias [30]. Despite these limitations, we believe that our results are important and highlight important changes in a very special population, from Lazio, Italy. Our data may support other researchers to understand the sequelae of lockdowns and help in future pandemic planning.

## 5. Conclusions

The aim of this study was to evaluate the potential impact of lockdown periods on eating habits and health risk factors through the use of a multi-topic questionnaire. Our results suggest that, overall, participants’ food preferences improved slightly (e.g., participants consumed more raw vegetables, more cereals, and fewer sugary drinks). We propose that the increased time spent at home allowed participants more freedom and flexibility in preparing fresh foods. It is conceivable that regular office workers may lack the time to cook fresh meals. Additionally, we highlighted that participants in our sample ate out less frequently, especially at lunchtime. The reduced exposure to snack bars or vending machines (which are frequently used to sell snacks, fruit juices, and soft drinks) may also have contributed to improved eating behaviors. Moreover, physical activity in the morning hours increased substantially. Again, greater flexibility and a more liberal day schedule may have contributed to the increased morning physical activity. However, in light of the cross-sectional nature of our study, this assumption requires further investigation. Ultimately, we also highlighted several negative aspects, e.g., an increase in reported difficulties in falling asleep, probably as a consequence of pandemic-induced (social) distress and discomfort. Additional trials are warranted to examine whether our results apply to other populations.

## Figures and Tables

**Table 1:** Body composition and other characteristics of study participants.

		TOTAL (*n* = 118)
Female	% (*n*)	72% (85)
Smokers	% (*n*)	12.7% (15)
Height	m	165.9 ± 7.8
Weight	kg	72.6 ± 15.9
FM	kg	22.2 ± 9.6
FFM	kg	48.2 ± 9.7
Type 2 Diabetes Mellitus	% (*n*)	5.9% (7)
Hypertension	% (*n*)	19.5% (23)
Dyslipidemia	% (*n*)	32.2% (38)
Mean physical activity	(METs/wk)	7.8 ± 8.4

Data are expressed as means ± SD. Abbreviations—SD: standard deviation; *n*: number of participants; FM: fat mass; FFM: fat-free mass; MET: metabolic equivalent. MET is defined as the amount of oxygen consumed while sitting at rest and is equal to 3.5 mL O_2_ per kg body weight x min.

**Table 2:** Consumption frequency differences before and after COVID-19 lockdowns.

		Before (*n* = 118)		After (*n* = 118)		Difference	*p*
		*n*	%	*n*	%	%	
Cow’s Milk	No	36	30.5	34	28.8	−1.7	0.577
	Rarely	15	12.7	16	13.6	0.9	
	Yes	67	56.8	68	57.6	0.8	
Low-Fat Low-Sugar Yo-Gurt	No	25	21.2	29	24.6	3.4	0.949
	Rarely	29	24.6	21	17.8	−6.8	
	Yes	64	54.2	68	57.6	3.4	
Vegetable Drinks (e.g., Soy Milk)	No	56	47.5	54	45.8	−1.7	0.235
	Rarely	23	19.5	18	15.3	4.2	
	Yes	39	33.1	46	39	5.9	
Whole Grain Food	No	9	7.6	7	5.9	−1.7	0.088
	Rarely	14	11.9	7	5.9	−6	
	Yes	95	80.5	104	88.1	7.6	
Cereals (e.g., Spelt, Barley)	No	22	18.6	10	8.5	−10.1	0.008
	Rarely	17	14.4	18	15.3	0.9	
	Yes	79	66.9	90	76.3	9.4	
Cooked Vegetables	No	0	0	2	1.7	1.7	0.475
	Rarely	8	6.8	7	5.9	−0.9	
	Yes	110	93.2	109	92.4	−0.8	
Raw Vegetables	No	18	15.3	3	2.5	−12.8	<0.001
	Rarely	7	5.9	6	5.1	−0.8	
	Yes	93	78.8	109	92.4	13.6	
Fruits	No	1	0.8	4	3.4	2.6	0.439
	Rarely	10	8.5	7	5.9	−2.6	
	Yes	107	90.7	107	90.7	0	
Legumes	No	1	0.8	5	4.2	3.4	0.034
	Rarely	6	5.1	10	8.5	3.4	
	Yes	111	94.1	103	87.3	−6.8	
Eggs	No	6	5.1	4	3.4	−1.7	1
	Rarely	10	8.5	14	11.9	3.4	
	Yes	102	86.4	100	84.7	−1.7	
Fish	No	6	5.1	13	11	5.9	0.106
	Rarely	16	13.6	11	9.3	−4.3	
	Yes	96	81.4	94	79.9	−1.5	
Fresh Cheeses	No	19	16.1	13	11	−5.1	0.195
	Rarely	4	3.4	8	6.8	3.4	
	Yes	95	80.5	97	82.2	1.7	
White Meat	No	10	8.5	13	11	2.5	0.471
	Rarely	11	9.3	10	8.5	−0.8	
	Yes	97	82.2	95	80.5	−1.7	
Red Meat	No	18	15.3	19	16.1	0.8	0.977
	Rarely	24	20.3	22	18.6	−1.7	
	Yes	76	64.4	77	65.3	0.9	
Processed Meat (e.g., Prosciutto)	No	9	7.6	9	7.6	0	0.21
	Rarely	5	4.2	12	10.2	6	
	Yes	104	88.1	97	82.2	−5.9	
Nuts	No	7	5.9	5	4.2	−1.7	0.821
	Rarely	2	1.7	6	5.1	3.4	
	Yes	109	92.4	107	90.7	−1.7	
Tofu	No	70	59.3	69	58.5	−0.8	0.718
	Rarely	26	22	31	26.3	4.3	
	Yes	22	18.6	18	15.3	−3.3	
Dark Chocolate (cocoa > 75%)	No	19	16.1	16	13.6	−2.5	0.941
	Rarely	6	5.1	11	9.3	4.2	
	Yes	93	78.8	91	77.1	−1.7	

Data are expressed as means ± SD. Abbreviations—SD: standard deviation; *n*: number of subjects.

**Table 3:** Differences in drinking habits before and after lockdowns.

		Before (*n* = 118)		After (*n*= 118)		Difference	*p*
		*n*	%	*n*	%	%	
How Many Liters of Water Do You Drink per Day on Average?	0.0	4	3.4	0	0	−3.4	<0.001
	1.0	64	54.2	47	39.8	−14.4	
	2.0	42	35.6	60	50.8	15.2	
	3.0	7	5.9	10	8.5	2.6	
	4.0	1	0.8	1	0.8	0	
How Many Sugary Drinks or Added Sugar Do You Consume per Day on Average?	0.0	47	39.8	71	60.2	20.4	<0.001
	1.0	23	19.5	18	15.3	−4.2	
	2.0	17	14.4	15	12.7	−1.7	
	3.0	14	11.9	7	5.9	−6	
	4.0	10	8.5	5	4.2	−4.3	
	5.0	2	1.7	1	0.8	−0.9	
	6.0	3	2.5	1	0.8	−1.7	
	7.0	2	1.7	0	0	−1.7	
How Many Times Do You Consume Alcoholic Beverages in a Week?	0.0	50	42.4	47	39.8	-2.6	0.235
	1.0	35	29.7	32	27.1	−2.6	
	2.0	19	16.1	16	13.6	−2.5	
	3.0	3	2.5	9	7.6	5.1	
	4.0	3	2.5	6	5.1	2.6	
	5.0	2	1.7	2	1.7	0	
	6.0	2	1.7	6	5.1	3.4	
	7.0	4	3.4	0	0	−3.4	
Do You Like Salty (1) or Sweet (10)?	1.0	10	8.5	10	8.5	0	0.279
	2.0	5	4.2	2	1.7	−2.5	
	3.0	9	7.6	11	9.3	1.7	
	4.0	4	3.4	8	6.8	3.4	
	5.0	33	28	25	21.2	−6.8	
	6.0	9	7.6	8	6.8	−0.8	
	7.0	8	6.8	10	8.5	1.7	
	8.0	19	16.1	14	11.9	−4.2	
	9.0	8	6.8	11	9.3	2.5	
	10.0	13	11	19	16.1	5.1	

Data are expressed as means ± SD. Abbreviations—SD: standard deviation; n: number of subjects. Participants drank more water (2 L per day 35.6% “before” vs. 50.8% “after”; *p* < 0.001) and fewer sugar-sweetened beverages (0 drinks per day: 39.8% “before” vs. 60.2% “after”; *p* < 0.001).

**Table 4:** Meals, sleep habits, and sports frequency differences before and after lockdowns.

			Before (*n* = 118)		After (*n* = 118)		Difference (%)	*p*
			*n*	%	*n*	%	%	
How Many Times Do You Eat a Day?		2.0	4	3.4	3	2.5	−0.9	0.143
		3.0	24	20.3	18	15.3	−5	
		4.0	37	31.4	32	27.1	−4.3	
		5.0	38	32.2	52	44.1	11.9	
		6.0	13	11.0	11	9.3	−1.7	
		7.0	2	1.7	2	1.7	0	
When Are You More Hungry during the Day?		When I Wake Up	14	11.9	13	11.0	−0.9	0.796
		During The Morning	34	28.8	28	23.7	−5.1	0.201
		In The Afternoon	22	18.6	25	21.2	2.6	0.532
		Before Dinner	40	33.9	39	33.1	−0.8	0.869
		After Dinner	6	5.1	7	5.9	0.8	0.739
		I’m Always Hungry	2	1.7	6	5.1	3.4	0.157
Do You Eat Fast?	No		35	29.7	31	26.3	−3.4	0.371
	Yes		83	70.3	87	73.7	3.4	
Do You Snack between Meals?	No		24	20.3	24	20.3	0	1
	Yes	Yes, before Lunch	11	9.3	12	10.2	0.9	0.782
		Yes, During The Afternoon	36	30.5	33	28	−2.5	0.639
		Yes, Before Dinner	33	28	30	25.4	−2.6	0.639
		Yes, After Dinner	14	11.9	19	16.1	4.2	0.317
Do You Eat Out at Meals?	No		29	24.6	57	48.3	23.7	<0.001
	Yes	I Often Eat at Restau-Rants for Dinner	10	8.5	5	4.2	−4.3	0.166
		At Lunch I Eat at Company Canteen	19	16	8	6.8	−9.2	0.012
		I Eat Lunch at Work or Bring It From Home	44	37.3	39	33.1	−4.2	0.384
		At Lunch I Eat in a Diner or Restaurant	16	13.6	9	7.6	−6	0.09
Do You Ever Eat Distracted or Not at the Table?	No		50	42.4	47	39.8	−2.6	0.647
	Yes		68	57.6	71	60.2	2.6	
Do You Ever Miss Meals?	No		89	75.4	95	80.5	5.1	0.201
	Yes (Total)		29	24.6	23	19.5	−5.1	
		Yes, I Have No Time	4	3.4	1	0.8	−2.6	0.18
		Yes, For No Reason	14	11.9	14	11.9	0	1
		Yes, For Craving Before Meals	3	2.5	1	0.8	−1.7	0.317
		Yes, I Always Skip Breakfast	8	6.8	7	5.9	−0.9	0.739
Do You Happen to Eat Uncontrollably Even if You’re Not Hungry?	No		27	22.9	33	28.0	5.1	0.335
	Yes (Total)		91	77.1	85	72.1	-5	
		Yes, Every Day	8	6.8	6	5.1	−1.7	
		Yes, Infrequent (1/month)	49	41.5	44	37.3	−4.2	
		Yes, Often (>1/Week)	34	28.8	35	29.7	0.9	
Do You Eat Differently on the Weekend?	No		22	18.6	30	25.4	6.8	0.131
	Yes (Total)							
		Yes, I Cook More Elaborate Meals	31	26.3	19	16.1	−10.2	0.04
		Yes, I Eat More at Home	32	27.1	36	30.5	3.4	0.564
		Yes, I Eat at Restaurants	33	28.0	33	28.0	0	1
Do You Wake Up to Eat at Night?	No		114	96.6	114	96.6	0	0.739
	Yes (Total)							
		Every Day	0	0.00	0	0.00	0	
		Infrequent (1/month)	1	0.8	2	1.7	0.9	
		Often (>1/week)	3	2.5	2	1.7	−0.8	
Do You Sleep Well at Night?	No							
		At Lunch I Eat in a Diner or Restaurant	12	10.2	20	16.9	6.7	0.033
		I Wake Up a Lot Sooner Than I Would Like	14	11.9	19	16.1	4.2	0.197
		I Wake Up Several Times During the Night	39	33.1	34	28.8	−4.3	0.317
	Yes (Total)		53	44.9	45	38.1	−6.8	0.144
Do You Play a Sport (at Least 5 Hours/Week)?	No		51	43.2	46	39.0	−4.2	0.317
	Yes		67	56.8	72	61.0	4.2	
When Do You Play Sport?		Before Breakfast	4	3.4	10	8.5	5.1	0.083
		In The Morning	15	12.7	26	22	9.3	0.016
		Before Lunch	8	6.8	4	3.4	−3.4	0.206
		In The Afternoon	18	15.3	18	15.3	0	1
		Before Dinner	23	19.5	15	12.7	−6.8	0.144
		After Dinner	1	0.8	0	0	−0.8	0
How Many Hours of Sports Do You Play per Week?		<5	47	39.8	52	44.1	4.3	0.497
		>10	1	0.8	1	0.8	0	
		0	52	44.1	47	39.8	−4.3	
		5–10	18	15.3	18	15.3	0	
Weekly METs		<5	53	44.92	49	41.53	−3.39	0.581
		6–10	22	18.64	31	26.27	7.63	
		11–15	16	13.56	16	13.56	0	
		16–20	19	16.1	12	10.17	−5.93	
		21–25	3	2.54	8	6.78	4.24	
		26–30	4	3.39	2	1.69	−1.7	
		>31	1	0.85	0	0	−0.85	

MET: metabolic equivalent. MET is defined as the amount of oxygen consumed while sitting at rest and is equal to 3.5 mL O_2_ per kg body weight x min.

## Data Availability

The datasets generated during and/or analyzed during the current study are available from the corresponding author on reasonable request.

## Supplementary Materials

The following are available online at https://www.mdpi.com/article/10.3390/nu13124522/s1, Table S1: Jobs performed by study subjects.
