# Supplementary material for: Changes in Eating Habits and Physical Activity after COVID-19 Pandemic Lockdowns in Italy
Source: Nutrients. 2021 Dec 17;13(12):4522. doi: 10.3390/nu13124522 (PMC8708956; doi:10.3390/nu13124522)
Supplement: Supplementary file 1 [file nutrients-13-04522-s001.zip › nutrients-1485069-supplementary.pdf]

Table S1. Different jobs of study subjects.

**Type of Job**	***n***
Architect	3
Artist	1
Bartender	1
Biologist	3
Cleaner	1
Computer Technician	3
Consultant	1
Dressmaker	1
Driver	1
Engineer	1
Farmer	1
Gym Worker	1
Homemaker	7
Ice Cream Seller	1
Fitness Instructor	1
Lawyer	2
Medical Doctor	1
Office Worker	52
Orthodontist	1
Physiotherapist	2
Policeman	1
Professor/Teacher	5
Psychotherapist	1
Researcher	3
Restorer	1
Retired	8
Sales Agent	1
Soldier	1
Student	7
Theater Scenographer	1
Unemployed	4
